# Association between quality of life and prognosis of candidate patients for heart transplantation: a cross-sectional study*

**DOI:** 10.1590/1518-8345.2602.3054

**Published:** 2018-10-11

**Authors:** Vanessa Silveira Faria, Ligia Neres Matos, Liana Amorim Correa Trotte, Helena Cramer Veiga Rey, Tereza Cristina Felippe Guimarães

**Affiliations:** 1 Hospital Pró Cardíaco, Heart Center, Rio de Janeiro, RJ, Brazil.; 2 Universidade Federal do Rio de Janeiro, Escola de Enfermagem Anna Nery, Rio de Janeiro, RJ, Brazil.; 3 Instituto Nacional de Cardiologia, Coordenação de Ensino e Pesquisa, Rio de Janeiro, RJ, Brazil.

**Keywords:** Heart Failure, Quality of Life, Heart Transplantation, Prognosis, Ambulatory Care, Adult

## Abstract

**Objective:**

to verify the association between the prognostic scores and the quality of life of candidates for heart transplantation.

**Method:**

a descriptive cross-sectional study with a convenience sample of 32 outpatients applying to heart transplantation. The prognosis was rated by the Heart Failure Survival Score (HFSS) and the Seattle Heart Failure Model (SHFM); and the quality of life by the Minnesota Living With Heart Failure Questionnaire (MLHFQ) and the Kansas City Cardiomyopathy Questionnaire (KCCQ). The Pearson correlation test was applied.

**Results:**

the correlations found between general quality of life scores and prognostic scores were (HFSS/MLHFQ r = 0.21), (SHFM/MLHFQ r = 0.09), (HFSS/KCCQ r = -0.02), (SHFM/KCCQ r = -0.20).

**Conclusion:**

the weak correlation between the prognostic and quality of life scores suggests a lack of association between the measures, i.e., worse prognosis does not mean worse quality of life and the same statement is true in the opposite direction.

## Introduction

The availability of solid organs for transplantation is a problem worldwide^1^ ^-^ ^4^. There had been an expressive increase in the number of cardiac transplantations (CT) in the world until the mid-1990s. Since then, due to improvements in the clinical management of heart failure (HF) and the inherent limitation of donors, the number of CT remains stable: 4,000 to 5,000^5^. In Brazil, in 2016, of the 631 patients entered in the CT queue, 145 died before receiving a heart, with only 357 CT being performed, which reaches 1.7 transplants per million population^6^. These facts reinforce the need for an accurate indication for CT, considering the risk stratification of the patients and the patient’s desire to transplant. 

In this context, studies have described the prognostic scores in HF as well-used and accurate measures to stratify risk^7^ ^-^ ^8^ and when associated to the peak of oxygen consumption (VO2) can help the indication of transplantation, according to the suggestion of the International Society for Heart and Lung Transplantation - ISHLT^9^, whereas the specific instruments of Quality of Life (QoL) have shown to be accurate in assessing QoL in patients with HF^10^ ^-^ ^11^.

Besides, scholars^12^ ^-^ ^14^ recommend that nursing progresses in research practices to evaluate outcomes such as QoL, prognosis and readmission in patients with advanced HF and transplant candidates, as well as after CT and clinical follow-up.

Therefore, as the improvement of QoL, in addition to increased survival, is one of the objectives to be achieved with the indication of the CT, and as HF has an impact on QoL, besides as a poor prognosis, this article aims to check the association between the prognostic scores and the QoL of candidates for CT.

## Method

This is a cross-sectional study delineated by a non-probabilistic or convenience sample, delimited initially by all the adult patients listed and being prepared for CT of the National Institute of Cardiology (INC) in Rio de Janeiro.

Data were collected from March to August 2016. Inclusion criteria were outpatient candidates for HT; being 18 years of age or over; having performed ergospirometry. Exclusion criteria were patients who have been admitted during data collection without the possibility of hospital discharge; diagnosis of psychiatric illness; incomplete medical records regarding the data necessary to classify prognostic scores.

**Figure 1:**
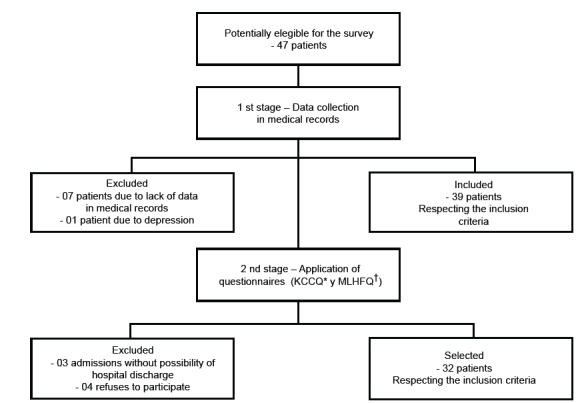
Scheme for the selection of research subjects

During the study period, 47 patients were potentially eligible and of these 32 patients were selected, as described in Figure 1.

*KCCQ - Kansas City Cardiomyopathy Questionnaire; †MLHFQ - Minnesota Living With Heart Failure Questionnaire.

Data collection was performed in the outpatient clinic of the INC, in two stages.

**Figure 2:**
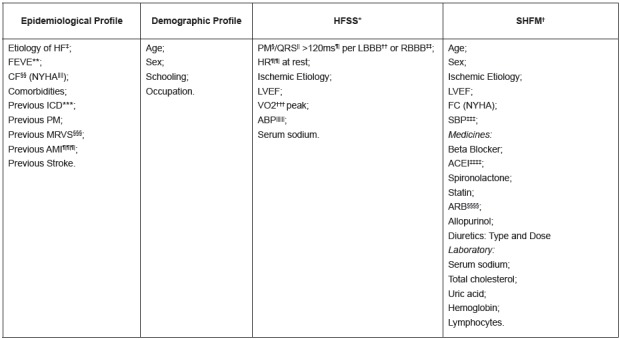
Variables collected in medical records

The first stage involved the data collection in medical records. The schedule of the certified physician for CT was used as a guide to identify the patients who were candidates for CT and to collect information on patients’ sociodemographic and clinical profile, as well as data for the classification by the Heart Failure Survival Score (HFSS) and the Seattle Heart Failure Model (SHFM), described in Figure 2.

*HFSS - Heart Failure Survival Score; †SHFM - Seattle Heart Failure Model; ‡HF - Heart Failure; §PM - Pacemaker; ||QRS - Ventricular depolarization; ¶ms - milliseconds; **LVEF - Left-Ventricular Ejection Fraction; ††LBBB - Left Bundle-Branch Block; ‡‡RBBB - Right Bundle-Branch Block; §§FC - Functional class; |||||NYHA - New York Heart Association; ¶¶HR - Heart Rate; ***ICD - Implantable Cardioverter-Defibrillator; †††VO2 - O2 consumption; ‡‡‡SBP - Systolic blood pressure;; §§§MRVS - Myocardial Revascularization Surgery; ||||||ABP - Average Blood Pressure; ¶¶¶AMI - Acute Myocardial Infarction; ‡‡‡‡ACEI - Angiotensin-Conversting Enzyme Inhibitor; §§§§ARB - Angiotensin-Receptor Blocker.

For the second phase of this research, a pilot test was carried out with the application of three questionnaires from the Minnesota Living Heart Failure Questionnaire (MLHFQ) and the Kansas City Cardiomyopathy Questionnaire (KCCQ), and we found that patients were unable to answer them alone, which can be explained by the schooling that ranged from elementary to higher education in this sample. For this reason, the interview method was chosen for this phase, and therefore, it was performed after the medical consultation. The four patients who missed the consultations were contacted via telephone for a new appointment, of whom two refused to participate and two answered the questionnaires at the next appointment.

The research instruments used were SHFM, HFSS, KCCQ and MLHFQ. The SHFM consists of 20 variables divided into clinical (age, sex, New York Heart Association - NYHA Functional Class - FC, weight, Left-Ventricular Ejection Fraction - LVEF, systolic blood pressure), medications (angiotensin-converting enzyme inhibitor - ACEI, beta-blocker-BB, angiotensin-receptor blocker - ARB, statin, allopurinol, aldosterone antagonist and type-specific diuretics), laboratory data (hemoglobin, lymphocytes, uric acid, total cholesterol, serum sodium) and Resynchronization Therapy (CRT) or Implantable Cardioverter-Defibrillator (ICD)^15^.

The HFSS is composed of six variables calculated by the following formula^16^:

*HFSS = [(0.0216 x resting heart rate) + (-0.0255 x mean systemic arterial pressure) + (-0.0464 x left-ventricular ejection fraction) + (-0.0470 x serum sodium) + (-0.0546 x oxygen consumption during maximal exercise) + (0.6083 x presence of intraventricular conduction defect) + (0.6931 x presence of coronary disease)]*

The MLHFQ^17^ is composed of 21 questions divided by two dimensions (physical and emotional) and total score. The total score is calculated with the sum of the questions ranging from 0 to 105, in which the higher the score, the worse the QoL.

And the KCCQ^18^ is composed of 15 questions, with 23 items, organized in five dimensions: Physical limitation; Symptoms (frequency/severity/stability); Quality of life, Self-care; and Social limitation. The result of the score ranges from 0 to 100, in which the higher the score, the better the QoL.

The collected data were compiled and processed by the Microsoft Excel® software of the Microsoft Office® package and the Statistical Package for Social Sciences (SPSS) 24 software, divided in three steps. The Shapiro-Wilk test was used to assess whether the sample distribution was normal.

In the first stage, simple descriptive statistics was performed to present the sociodemographic and clinical profile of the sample. The second step also consisted of a descriptive analysis of the prognostic scores (HFSS and SHFM) and the QoL scores (MLHFQ and KCCQ). 

The third step consisted of correlation analyzes between the two prognostic scores, with the QoL scores. The Pearson correlation coefficient (r) was used which presupposes a linear correlation between quantitative variables. For this study, we used the reference that categorizes the correlation at r = 0.10 to 0.30 (weak); r = 0.40 to 0.6 (moderate); r = 0.70 to 1 (strong).

The present study was approved by the Ethics and Research Committee of the hospital where the research was carried out under approval number 51348515.0.0000.5272, and all the participants signed the Informed Consent Form. 

## Results

Table 1 presents the sociodemographic and clinical characteristics of the participants.

**Table 1:** Sociodemographic and clinical characteristics of the sample (n = 32). Rio de Janeiro/RJ, Brazil, 2017

Patients’ Characteristics	n = 32	%
*Sex*		
Female	14	43.75%
Male	18	56.25%
*Age group (years)*		
25-45	10	31.25%
46-55	10	31.25%
56-65	12	37.50%
*Occupation*		
Retired due to disability	18	56.24%
Sick leave by social security	7	21.88%
Others	7	21.88%
*Schooling*		
Elementary School (1st to 5th year)	8	25.00%
Secondary School (6th to 9th grade)	6	18.75%
High school	11	34.37%
Higher education	7	21.88%
*Etiology*		
Idiopathic	11	34.38%
Others	8	25.00%
Ischemic	7	21.88%
Valvar	4	12.50%
Chagasic	2	6.25%
*Clinical Data*		
SAH*	12	37.50%
Type II DM^†^	5	15.63%
AF^‡^	9	28.13%
DLP^§^	6	18.75%
FC^||^ NYHA^¶^ III	26	81.25%
FC NYHA IV	6	18.75%
ICD**	9	28.13%
PM^††^	3	9.38%
Previous AMI^‡‡^	9	28.13%
Previous stroke^§§^	8	25.00%
Previous VS^||||^	5	15.63%
Previous MRVS^¶¶^	3	9.38%

*SAH - Systemic Arterial Hypertension; †DM - Diabetes Mellitus; ‡AF - Atrial fibrillation; §DLP - Dyslipidemia; ||FC - Functional Class; ¶NYHA - New York Heart Association; **ICD - Implantable Cardioverter-Defibrillator; ††PM - Pacemaker, ‡‡AMI - Acute Myocardial Infarction; ||||VS - Valvar Surgery; ¶¶MRVS - Myocardial Revascularization Surgery.

When classified by the HFSS, 89.2% of the patients were described as medium and low risk for mortality in one year ahead, however, when classified by the SHFM, 90.6% were described as medium and high risk for mortality in one year ahead.

The mean QoL scores of the participants by the MLHFQ and KCCQ questionnaires are described in Table 2.

**Table 2:** Classification of the quality of life of participants by the Kansas City Cardiomyopathy Questionnaire and the Minnesota Living With Heart Failure Questionnaire, divided by dimensions (n = 32). Rio de Janeiro/RJ, Brazil, 2017.

Quality of Life Instruments	Mean	Confidence interval	Standard deviation
KCCQ*			
Symptom Frequency	64.00	± 9.37	± 27.04
Symptom Severity	65.36	± 8.27	± 23.86
Symptom Total Score	64.68	± 8.36	± 24.14
Quality of life	44.01	± 7.56	± 21.82
Social Limitation	43.42	± 8.76	± 25.30
Clinical Score	53.13	± 7.92	± 22.85
Overall Score	48.43	± 6.90	± 19.92
*MLHFQ* ^†^			
Overall Score	48.41	± 8.32	± 24.00
Physical Dimension	20.97	± 4.01	± 11.57
Emotional Dimension	10.56	± 1.99	± 5.75

*KCCQ - Kansas City Cardiomyopathy Questionnaire; †MLHFQ - Minnesota Living With Heart Failure Questionnaire.

The Pearson correlation matrix between the general scores of quality of life instruments and the prognostic tools showed the following absolute values: HFSS x MLHFQ - 0.21; HFSS x KCCQ = 0.02; SHFM x MLHFQ = 0.09; and SHFM x KCCQ - 0.20.

When analyzing the relationships between individual prognostic scores (HFSS and SHFM) with distinct quality of life scores (MLHFQ and KCCQ), we found in all cases a weak correlation, with the highest value found for r = 0.21, which suggests that there is no association between the two prognostic scores with the two QoL measurement instruments, that is, patients with worse prognosis may present good quality of life and vice versa. 

## Discussion

The weak correlation between the prognostic scores and the QoL scores found in this study suggests that the patient’s perception, measured by QoL, as well as the prognostic score are a complementary measure to be used in clinical practice to aid the indication of CT.

No studies were found in the literature that associate prognostic scores with specific QoL instruments in HF, however one study evaluated the relationship between SHFM and a generic QOL instrument^19^. Also, some studies have discussed the impact on the mortality of the specific instruments that measure QOL in HF^20^ ^-^ ^22^.

One study longitudinally evaluated the relationship between SHFM and the health status valuation measured by the generic instrument EuroQol 5D (EQ-5D). Through a linear regression, they evaluated 2,331 patients with a 2.5-year follow-up, with FC (NYHA) II to IV, LVEF ≤ 35%, showing that the increase of 1 unit in SHFM decreased by 0.03 points the EQ-5D in the baseline assessment and that each year that the SHFM increased in one point, the EQ-5D decreased 0.006 points. These results showed that patients with high mortality risk had significantly lower EQ-5D and had higher rates of decline over time^19^.

Regarding the impact on mortality, one study followed 8,443 patients with reduced LVEF for 4.8 months and annually to assess the association of KCCQ with mortality in a randomized clinical trial comparing the use of enalapril with a new class of drugs, namely the LCZ696, which is a medicine composed of two complementary pharmacological agents. One of them, valsartan is a direct blocker of ARB, and the other is an inhibitor of neprilysin, an enzyme responsible for the degradation of endogenous vasodilator peptides, such as bradykinin, natriuretic peptides and calcitonin gene-related peptide, among others. And it concluded that KCCQ is associated with survival. Changes in QoL were better in patients treated with LCZ696 compared to enalapril, with consistency in most domains. These findings suggest that LCZ696 leads to better QoL^20^.

Another study observed patients for three years, measuring B-type natriuretic peptide (BNP), and used the overall well-being evaluated by Cantril’s Ladder of Life, the MLHFQ to evaluate QoL and the Medical Outcome Study 36-item General Health Survey (RAND36) as a generic instrument and concluded that QoL is an independent predictor for survival^21^.

In addition, a systematic review and a meta-analysis of prospective cohorts with patients with stabilized HF and with follow-up of at least 1 month, published between 2002 and 2013, used KCCQ and MLHFQ to assess mortality and concluded that these instruments are significant mortality predictors besides the traditional risk factors^22^.

Whereas ISHLT^9^ ^)^ suggests the use of the HFSS or SHFM prognostic scores associated with VO2 peak to aid the indication to the CT, the difference in the risks found between the two scores in the same sample can be explained by the different variables considered by each score, such as the VO2 peak present in the HFSS, an important predictor for the indication of CT and absent in SHFM, as well as drugs such as BB, spironolactone and ICD, which improve the survival of this population, present in SHFM, but absent in the HFSS. Thus, SHFM was more reliable for classification of the prognosis in this sample.

Regarding the evaluation of QoL, the mean scores of the MLHFQ are in line with the study that dealt with QoL in patients with advanced HF in the CT queue that resulted in a mean of the total score of 40.61, of the physical dimension of 14.96 and of the emotional dimension of 7.70 ^23^. In the KCCQ, patients’ perception of QoL is similar to the study that evaluated the QoL of outpatients with FC III (NYHA): overall score (52.00), symptom total score (67.38), and symptom frequency score (67.00)^24^.

Although it is assumed that the advanced stage disease presents more symptoms, causes greater dysfunction and consequently is related to poorer quality of life and worse prognosis^25^ ^-^ ^26^, this may be true for an individual, but not necessarily it is the reality in a heterogeneous group of patients. 

Thus, even if a relationship between prognosis and quality of life can be established in larger samples, as has been the efforts of studies in this area, great individual variation should not be overlooked, since patients with the disease in similar stages may differentiate their symptoms and their limitations. In addition, non-prognostic QoL measurements can provide relevant information on opportunities to improve patient care^27^, especially in the case of indication for CT, which aims to improve survival and QoL^9^ ^,^ ^26^.

This research had some limitations, such as the size of the sample, data collection in a single center, the absence of information in the records for collection, as well as a scarce literature regarding the association of the specific instruments of quality of life with the scores of HF prognostics. 

We suggest verifying the correlation between the prognostic scores and the physical and emotional dimensions of the QoL questionnaires (MLHFQ and KCCQ). Another approach would be to verify causality between instruments. In addition to these issues, an important study would be on the applicability of these tools in clinical practice, such as the feasibility of implementation in the workflow, integration with the institution’s electronic records and studies on costs, allowing the infrastructure to collect data and analyze them.

## Conclusion

The weak correlation between the prognostic and QoL scores suggests the non-association between the scores, i.e., worse prognosis does not mean worse QoL and the opposite is also true. 

The evaluation of the association between the HFSS and SHFM prognostic scores with specific instruments of QoL (MLHFQ and KCCQ) in candidates for CT is important and necessary, and the present study contributed to the pioneering nature of this practice in Brazil and also made it when using the KCCQ in the Brazilian population.
